# Is There a Doctor on Board? We Have an Emergency!

**DOI:** 10.5704/MOJ.2311.017

**Published:** 2023-11

**Authors:** S Ibrahim, J Sathar

**Affiliations:** 1 Paediatric Orthopaedic Uni, UKM Specialist Children's Hospital, Kuala Lumpur, Malaysia; 2 Department of Haematology, Ampang Hospital, Ampang, Malaysia

Dear Editor,

September 16, 2023 was Malaysia Day and we were on a 3-hour flight from Makassar, Indonesia to Kuala Lumpur, Malaysia. Two hours into the flight, the cabin crew called for medical assistance – a passenger in the back row was ill. We answered the call and identified ourselves as doctors by presenting the invitation letters for our medical lectures in Makassar as we did not carry our hospital identification (ID) cards.

A 65-year-old male passenger in an aisle seat was in distress. He is diabetic and had taken his oral hypoglycaemic medications in the morning. Clinical examination showed him to be drowsy, cold and clammy with a thready pulse - the signs of hypoglycaemia. He felt better after a sweetened drink and insisted on walking to the lavatory, assisted by his wife. However, upon returning to his seat, he became drowsier and was slipping into a hypoglycaemic coma.

We informed the crew that this was a diabetic emergency and we needed to open the emergency medical kit. We found 3 intravenous (IV) cannulas of different sizes, 0.5 L of normal saline and 20 ml of 40% glucose. Our initial attempt to get an IV line on the dorsum of the hand was unsuccessful due to vibrations from the plane. Because of the dim cabin lighting, the crew used the light from a mobile phone during the procedure. We were able to insert the IV line in the antecubital vein in the next attempt (Fig. 1) and gave a bolus dose of glucose through the saline infusion (Fig. 2) just as the pilot announced the plane was preparing for landing.

**Fig 1:**
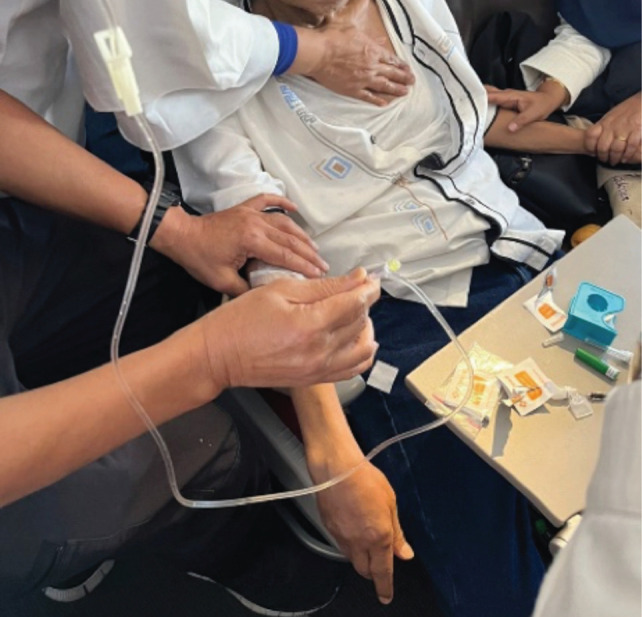
IV infusion of normal saline through the antecubital vein.

**Fig 2:**
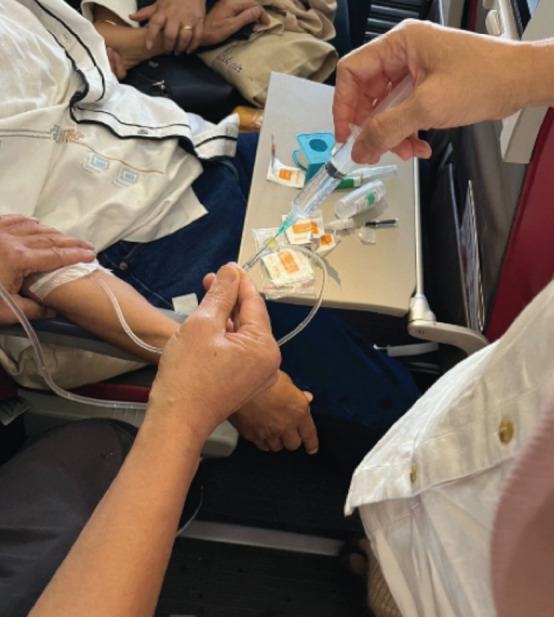
IV bolus dose 20 ml of 40% glucose.

The passenger swiftly recovered after the glucose infusion and declined further evaluation at a hospital. We had to complete and sign the medical incident report form before disembarking. A text message received the following day confirmed that the passenger was well and able to continue with his holiday in Malaysia.

We have been flying for over 40 years and this was the first time we faced an in-flight medical emergency. There are several salient aspects that we like to share from this experience.

We were the only medical staff to respond to the emergency on this flight. Malaysia does not have a Good Samaritan law and we have no legal obligation to volunteer but we are ethically bound to help. The Good Samaritan law protects people from legal repercussions when helping an ill person^1^. Some countries in Europe and Australia have laws that require doctors to respond in an emergency. However, in the USA, Canada, Singapore and the UK, there is no legal requirement for a doctor to volunteer during an in-flight emergency^2^.

We were able to remain calm and focussed even though we were in a cramped space observed by crew and fellow passengers. It was a challenge to search for the items in the emergency medical kit because they were not arranged in easily identifiable compartments. We now know that passenger planes carrying more than 100 passengers are required to have an emergency medical kit containing equipment and drugs for resuscitation^2^.

Doctors will be required to provide evidence of their medical credentials^3^. Since we did not have our hospital ID cards with us, we recommend that doctors store digital copies of their ID cards in their mobile phones for quick identification when travelling.

We thank the cabin crew for their assistance and for providing the photographs included in this letter.

## References

[1] Ashraf AI, Faiz N, Ariffin A (2017). Imposition of Good Samaritan Laws to Improve Professionalism among Medical Practitioners.. Intellect Discourse..

[2] Low RCH, How CH (2021). Responding to an in-flight medical emergency.. Singapore Med J..

[3] Smith LN (2008). An otolaryngologist's experience with in-flight commercial airline medical emergencies: three case reports and literature review.. Am J Otolaryngol..

